# Sub-cell scale features govern the placement of new cells by honeybees during comb construction

**DOI:** 10.1007/s00359-023-01632-y

**Published:** 2023-05-10

**Authors:** Vincent Gallo, Alice D. Bridges, Joseph L. Woodgate, Lars Chittka

**Affiliations:** Department of Biological and Experimental Psychology, School of Biological and Behavioural Sciences, Queen Mary University of London, London, E1 4NS UK

**Keywords:** Hexagon, Honeycomb, Stigmergy, Wax

## Abstract

Honeybee comb architecture and the manner of its construction have long been the subject of scientific curiosity. Comb is characterised by an even hexagonal layout and the sharing of cell bases and side walls, which provides maximised storage volume while requiring minimal wax. The efficiency of this structure relies on a regular layout and the correct positioning of cells relative to each other, with each new cell placed at the junction of two previously constructed cells. This task is complicated by the incomplete nature of cells at the edge of comb, where new cells are to be built. We presented bees with wax stimuli comprising shallow depressions and protuberances in simulation of features found within partially formed comb, and demonstrated that construction work by honeybee builders was influenced by these stimuli. The building of new cells was aligned to concave stimuli that simulated the clefts that naturally appear between two partially formed cells, revealing how new cells may be aligned to ensure proper tessellation within comb. We also found that bees built cell walls in response to edges formed by our stimuli, suggesting that cell and wall construction was specifically directed towards the locations necessary for continuation of hexagonal comb.

## Introduction

Honeybee comb is a double-sided sheet of tessellated, near-horizontal hexagonal cells formed from wax, with the pyramidal cell bases interlocking with those of the cells on the other side of the shared backplane; resulting in an offset of ½ a cell between the two sides (Graham 1993). The geometry and efficiency of this structure and the manner of its construction have inspired scientific curiosity for centuries, with the earliest analysis of honeycomb being attributed to Pappus of Alexandria, circa 320 CE. He observed that honeybees make “*honeycombs (with cells) all equal, similar and contiguous to one another… they have contrived this by virtue of a certain geometrical forethought…Bees, then, know just that the hexagon is greater than the square and the triangle and will hold more honey for the same expenditure of material*” (Heath 1921). Indeed, the hexagonal cells built by honeybees are more efficient than the round cells found in other species such as bumblebees, as this latter arrangement wastes material through duplication and increased space between cells (Gallo and Chittka 2018). The design of comb is so elegant that Darwin viewed it as a potential challenge to his theory of natural selection (1859). Natural theologians considered its regularity and optimisation to be proof of intelligent design: “*So that she has hit upon the very form which in every respect is the most advantageous and turns out to be on all grounds right as indeed we might well suppose when we recollect who is her Teacher*” (Brougham 1839). Ultimately, Darwin remedied these doubts by pointing out that some of the apparent complexity in comb building could emerge by relatively simple building rules and self-organisation. This has been the prevailing view ever since (Drory 1873; Stadelmann 1895; Silvestri 1902; Vogt 1911; Armbruster 1920), though the actual rules that guide the building bees remain poorly understood.

Self-organisation is a process by which the adherence to a small set of simple rules can produce a stable outcome that, at a macroscopic level, is both predictable and repetitive (Haken 1978; Camazine 2001). Stigmergy is a form of self-organisation where the rules remain simple, but the participants actively respond to local conditions (even if they themselves did not create them), and represents a potential mechanism to coordinate the activity of multiple actors towards a collective goal (Grassé 1960). Stigmergy has been proposed as a mechanism responsible for the coordination of activities within colonies of social insects (Bonabeau et al. 1999; Collignon and Detrain 2019). Stigmergy is a candidate mechanism behind the parallel blades of comb formed by honeybees (Hepburn and Whiffler 1991) and the pattern of cell use within a hive (Camazine 1991), but it is as yet unknown whether stigmergy also represents the coordinating mechanism that leads to the construction of each cell within honeycomb.

Patterns arising from simple, self-organised, mechanistic processes are, in fact, rather common in nature. In particular, hexagonal patterns often manifest in systems involving fluids that have reached equilibrium, such as equal-sized bubbles in foam, convection within a layer of fluid (Koschmieder and Pallas 1974; Korenić et al. 2020) and circulatory currents at the poles of Saturn (Fletcher et al. 2018). The common occurrence of hexagons may suggest that the construction of comb by honeybees is relatively straightforward and, potentially, a matter of self-organisation. However, the patterns that appear in these fluid systems are often uniform only at the centre, with heptagonal or pentagonal cells and other irregularities appearing closer to the edge, indicating that a non-uniform environment will give rise to an irregular layout. In nature, honeycomb construction begins with wax deposits on the underside of supporting structures, such as tree branches, rocky outcrops, or the upper surface of a cavity, all of which are distinctly non-uniform. Further complications are added by the bees themselves: for example, separate tongues of comb are constructed individually and in an uncoordinated manner but must ultimately be united. The wax from which comb is formed is also solid, meaning that the cells cannot slide over one another as in the fluid systems described above. This means that the final position of a cell is dependent mainly on its starting position, and hence, construction of each cell must be located correctly from the outset. Random cell placement is, therefore, unlikely to result in the regular, tessellated hexagon pattern observed in completed comb.

While measurements, descriptions, and analysis of the structure of completed cells and comb are plentiful (Hepburn and Whiffler 1991; Hepburn et al. 1991; Yang et al. 2021; Smith et al. 2021), nascent cells are less well characterised. This is largely due to difficulties in observing the process of comb building: even in an observation hive, both construction workers and the workpiece are typically covered by other bees. Huber (1814) provided the first description of the initial deposition and sculpting of wax that eventually formed two rows of cells: the first action being the removal of wax to enlarge a small indentation. The following step was the addition of wax to extend the dished area until *“…the diameter of the cavity was equal to that of an ordinary cell…*” whereupon he noted that wax was added to the periphery. Work progressed at multiple sites, allowing Huber to observe the conjunction of two nascent cells as “*two adjacent cavities… separated only by a common edge, formed from the gathering together of the wax particles drawn from their interior”* (1814). The initial stages of cell construction have also been modelled using a computer simulation of cell layout, which assumed that a cell base, a shallow dish, would be formed by expansion of an existing inter-cell cleft (Nazzi 2016).

**Fig. 1:**
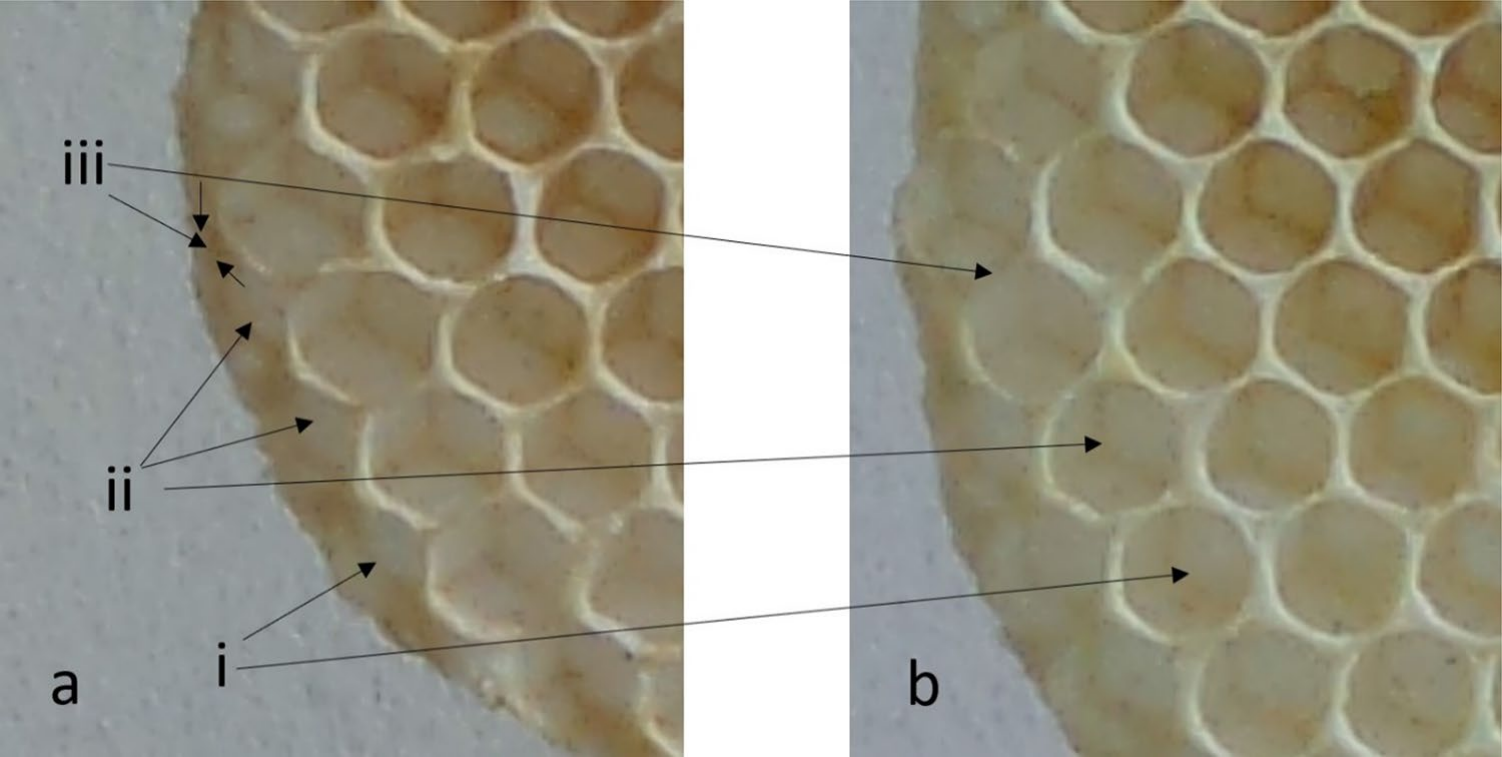
Construction guided by sub-scale features at the edge of honeycomb. (**a**) The edge of a section of comb at stage 1. (**b**) A subsequent photograph aligned to show the same section of comb after a further 4 h of construction. (**i**) A shallow depression has been expanded to become a cell. (**ii**) Two adjacent shallow depressions have both become cells with a wall dividing the cells lying orthogonal to the line connecting the two depressions. (**iii**) A wall dividing two new cells at the cusp of an existing cell wall

The goal of the present study was to determine how bees decide where to construct new cells and whether this would be influenced by shapes and features within the existing wax. Both Huber’s description and Nazzi’s model indicate that the presence of a concave site on the outer surface of existing cells, such as at the point where two cells meet, will trigger a reaction by builders to extend the depression. Hypothetically, and using the language of stigmergy, the local condition of a depression triggers a reaction to extend the concavity resulting in a nascent cell (Fig. 1i), and thus, an existing depression formed between two extant cells guides the location for a new cell to be built. At the edge of the comb, three cells will create two depressions, one either side of the central cell, with the result that a wall will be built mid-way between the two new cells and perpendicular to the line between their centres (Fig. 1ii). Additionally, the edge of the enlarged depression, at the point where the walls of the existing cell turn away, creates a condition that will trigger a new reaction to deposit wax around the edge. Eventually, those deposits will become a new cell wall (Fig. 1iii).

The assumption that cell construction will proceed according to these hypotheses led to three predictions, as follows:

1. A stimulus comprising a shallow depression will focus cell-formation activity leading to its expansion and wax deposition at its edges will lead to the eventual location of cell walls (Fig. 1i).
2. Two shallow depressions will both attract wax depositions at their rim, leading to a cell wall being constructed at the mid-way point between the two. The resulting wall will lie orthogonal to the line connecting the centre points (Fig. 1ii).
3. A stimulus in the form of a low wall creates a concavity and so will initiate the cell construction. The end of the ‘wall’ will be perceived as an edge, a horizon where the surface turns away, thus attracting wax deposition (Fig. 1iii). Two such stimuli joined at a V-shape will cause two cells to be formed, and both will result in a new wall being built at the cell intersection. The resulting wall will lie at an angle that bisects the stimulus “V”.

To test these predictions, we fashioned stimuli comprising wax forms where each was designed to trigger one of the predicted behaviours. We then placed the stimuli in hives, leaving the bees to build honeycomb upon them. These samples were inspected periodically and, when appropriate, the alignment and position of cell walls were measured for comparison with those of the stimuli.

## Materials and methods

### Hive handling and recording

#### Hives

Our studies were conducted from May to July 2020 and from June to July 2021 at an apiary in Reigate, England (51.23° N, 0.19° W), using three colonies of honeybees (*Apis mellifera*). The colonies were headed by locally reared queens and were housed in Modified British National hives comprising an open mesh floor and a single brood box containing 11 frames; including 10 conventional frames plus one to carry the experimental wax stimulus. All hives were configured in ‘warm’ alignment, that is with the frames set transverse to the entrance. The test frame was placed in each hive as the seventh frame from the front, at the edge of the brood area. Continual comb production was encouraged by the constant provision of ad libitum 1:1 sucrose solution (1.0 kg cane sugar in 1.0 l water).

### Preparation of stimuli

We created four different wax stimuli to investigate different elements of early stage comb building. Wax used to construct the experimental stimuli was recovered from hives within the same Reigate apiary. To create the stimuli, one face of a flat wooden form (75 × 40 mm) was coated by dipping into molten wax. Wax sheets of two thicknesses were produced by altering the number of immersions: three immersions produced sheets of 0.5–0.6 mm thickness, while six yielded sheets of 1.0–1.2 mm. These wax sheets, once cut into three pieces (25 × 40 mm) referred to henceforth as tabs, were held in place by adhesion to the top bar of an otherwise empty test frame and placed vertically within the hive. The face of each tab carried an adornment particular to each experiment, as detailed below.

**Fig. 2:**
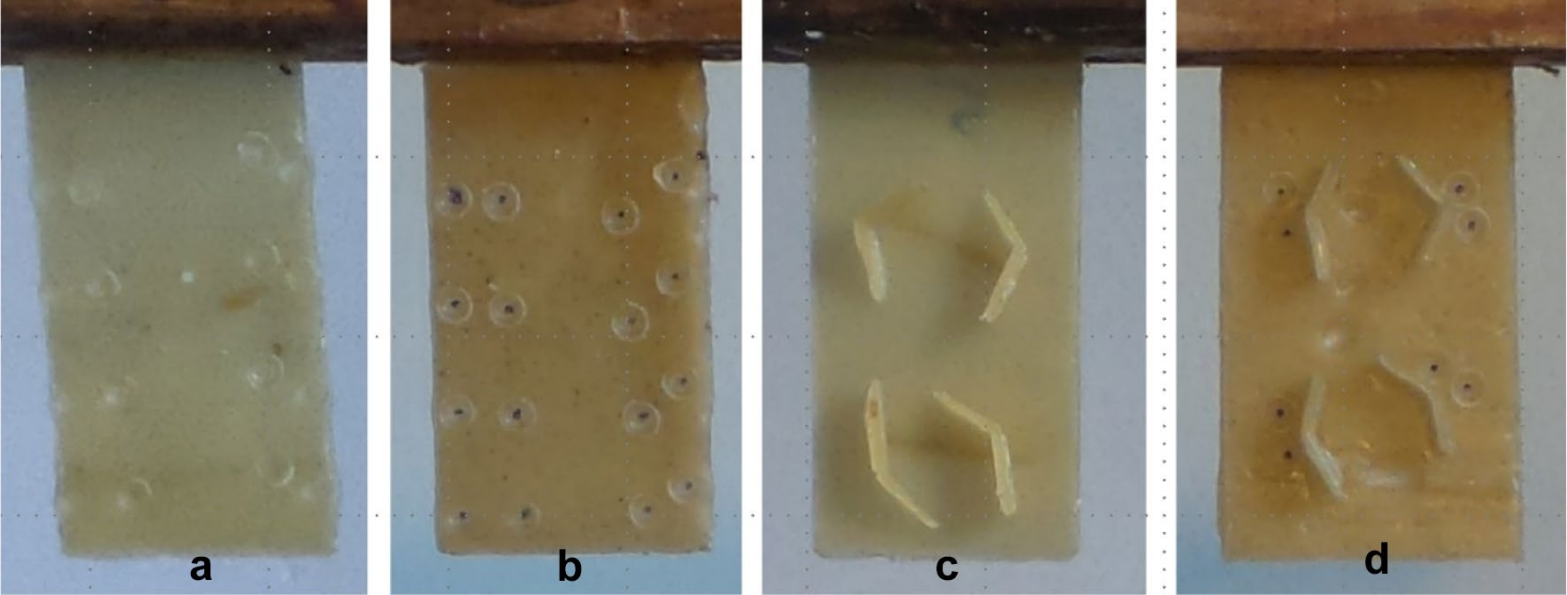
Four tabs carrying examples of the stimuli used in each experiment. These shapes were placed within the hives to allow comb to be built upon them. (**a**) Single depressions pressed into the wax tab, as used for experiment 1. (**b**) “Pairs of pits” comprising two depressions pressed into the wax tab in close proximity, as used for experiment 2. (**c**) “V-shapes” consisting of a ~ 2–3 mm high barrier welded onto the wax tab, as used for experiment 3. (**d**) “V-shapes”, as used in experiment 3, combined with “pairs of pits”, similar to those offered in experiment 2. The two stimuli shapes were offered together in experiment 4

***Experiment 1.*** Our first prediction stated that, when encountering a shallow dip in the wax, bees should initially deposit wax at the rim of the dip. Stimuli to test this prediction comprised shallow indentations which were pressed into one side of a tab using a 4 mm-diameter domed rod. The resulting indentations were ~ 0.25 mm deep and between 3 and 4 mm in diameter. If our prediction was correct, then the bees would focus construction at each pit to form a cell. The indentations were placed ad hoc (with 6–8 indentations per tab), ~ 10–15 mm from each other (Fig. 2a).

***Experiment 2.*** Our second prediction stated that, when presented with a stimulus that includes two small depressions, bees will construct two cells conjoined at a wall aligned to the common tangent between the two pits: i.e., orthogonal to a theoretical line connecting the pit centres. As above, pairs of shallow indentations were pressed into the wax, with 1–3 mm between each indentation. A total of six or eight pairs were pressed into each tab, with 10–15 mm between each pair (Fig. 2b). The orientation of each pit pair was ad hoc, albeit deliberately varied.

***Experiment 3.*** Our third prediction stated that bees will respond to a V-shaped barrier by constructing a cell on either side of the apex, conjoined at a wall aligned to the bisection of the barrier. A wax strip (2–3 mm in height and 0.5 mm in width), cut from the wax stock used to form the tabs, was folded to form a V-shape, and then welded onto the wax backplane, placed ad hoc (with four per tab), ~ 10–15 mm from each other (Fig. 2c). The angle and orientation of the ‘V’ was ad hoc, albeit deliberately varied.

***Experiment 4.*** Here, we combined the stimuli from experiments 2 and 3, offering a V-shaped barrier with a pair of pits pressed into the substrate close to the apex. Stimuli were constructed as described for Experiment 3, but a pair of shallow indentations were also pressed into the face of the backplane, with one on either side of the apex. The orientation of the V and that of the pit pair were manually, but deliberately, misaligned (Fig. 2d). This experiment was used to test whether cell construction would be guided by one stimulus more strongly than the other. If construction is preferentially guided by one stimulus, then wall alignment to the V-shape bisection will differ from its alignment to the pit common tangent by more than is expected by chance.

### Stimulus handling and construction time

**Fig. 3:**
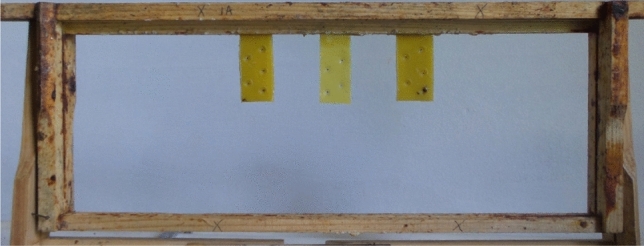
An example frame carrying stimuli-bearing tabs. The image shows a wooden frame carrying tabs prepared with single depressions, as used for experiment 1. The tabs were adhered to the top bar of the frame using molten wax, and the frame was placed within the brood region of a hive. Once within the hive, the bees built their comb upon the wax forms

The wax stimuli were positioned vertically and were wax-welded to the underside of the top bar of hive frames. Each frame carried three tabs spaced approximately 20 mm apart (Fig. 3). The frames were placed into honeybee hives at approximately 9 am, inspected and photographed after approximately 4 h and, if insufficient comb construction had occurred, they were reinserted and removed again at around 5 pm. Frames were not left in the hives overnight.

The frames carrying stimuli were photographed prior to being placed in the hives, and during each inspection. These photographs were used to analyse the progression of comb construction.

### Measurement methods

#### Recording and photography

Photography was performed in a room adjacent to the apiary, using natural daylight. Each frame was photographed while mounted on a jig that held it and the camera. The camera (Samsung Galaxy Camera 2 EK-GC200) yielded images of 4608 by 2592 pixels. The jig held the frames 390 mm from the camera, resulting in the frame (width 333 mm) occupying an image width of approximately 3000 pixels. Image resolution was therefore approximately 9 pixels per mm.

#### Photographic record analysis

Image manipulation was performed using custom software, *FormImageCompare*, written by the authors. This tool facilitates the alignment of images taken before and after a treatment, magnification, marking of features, and obtaining measurements, such as position, location, and angle from those marks. *FormImageCompare* was written in C +  + using Microsoft Visual Studio Community 2019: Version 16.7.2, Visual C +  + 2019, drawing on support from the library OpenCV:Version 3.3. This tool is available at https://github.com/VinceGalloQMUL/honeycombThesisRepo.

The photography jig provided a degree of consistency between photographs but was insufficient for direct comparison of wax features at the cell and cell wall scale. This limitation was overcome during the analysis by the alignment of features evident on the frame in both images. Using four such alignment points, the software could eliminate scale, displacement, rotation, or perspective change between the first and second photographs. Once the two images had been aligned, the position and shape of comb features could be compared with either the initial stimulus or the same feature at an earlier stage of construction.

## Experiment-specific measurements

### Experiment 1: initial deposition target (single pits)

To measure the degree of overlap between the rims of the seed pits and the eventual comb built on each wax panel, we analysed photographs of the initial and subsequent states, marking and recording the positions of the stimuli pit rims at the length of overlap between these and cell walls appearing in the subsequent image. Data for control samples were generated by measuring the overlap between virtual depressions and the constructed cell walls. Virtual depressions were computer generated, randomly located circles drawn on the photograph by the software, and for each, we marked the overlap of this line and cell walls.

**Fig. 4:**
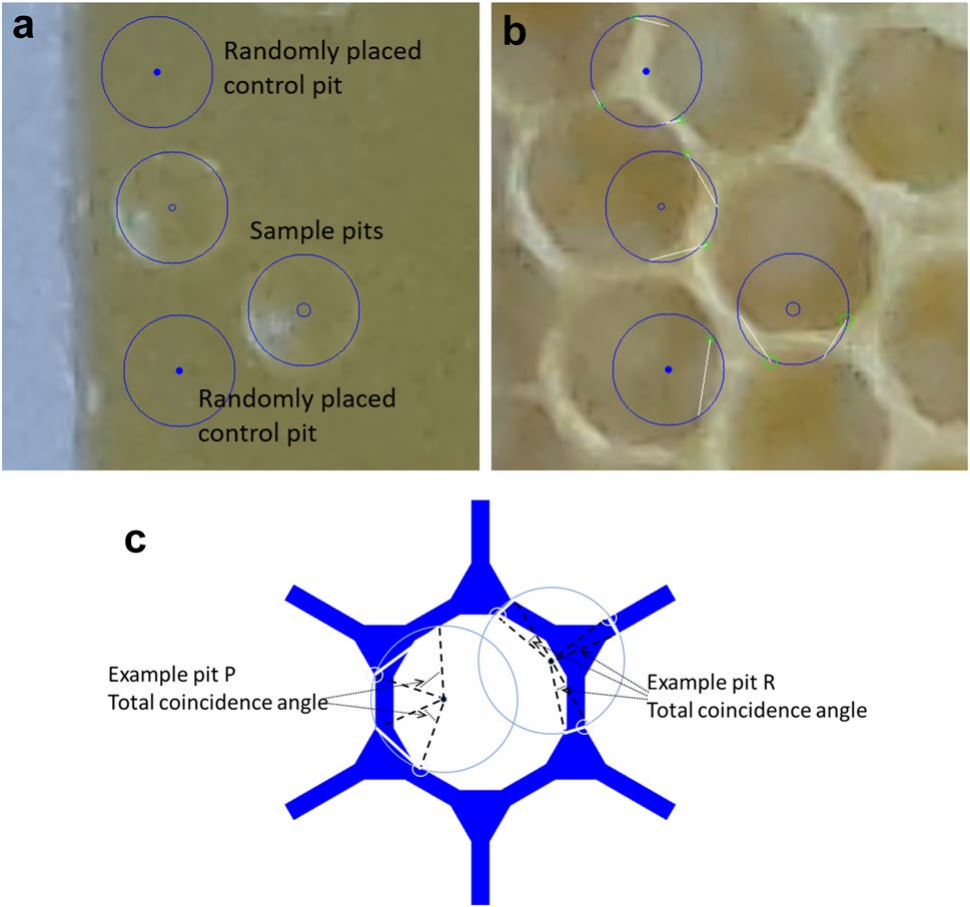
Pit rim to cell wall coincidence measurement. (**a**) Sample pits, visible as a depression in the wax, around which the software has drawn a circle to act as a measurement gauge. Also shown are two software-generated randomly placed virtual pits used to compile the control data set. (**b**) A view after the operator has marked the extent of overlap between the gauge circles and the cell walls. The operator clicked on the start and end of the overlap with a cell wall, causing the software to draw a chord (white line from the green circle) showing the extent of the overlap. (**c**) A diagrammatic representation of the measurements to be taken from the gauge and overlap marks. The metric, calculated and exported by the software, is the total angular overlap for each pit: it is larger for example pit P than for example pit R

Measurements of pit locations and the associated cell walls used the coordinates of markers placed manually on paired images. The software FormImageCompare:pitRim() accepts a user input mark at the centre of a stimulus depression, around which the software draws a circle scaled to the equivalent of 4.0 mm diameter. With the second image, which displayed the state of the tab after some comb had been built, the user could mark the start and end of any sections of the guide circle (representing the rim of the pit) overlapped with any walls that had been built (the overlap chord drawn in white by the software). Using the start and end of each overlap, the software calculated the total angular overlap between cell walls and a pit rim (Fig. 4).

Samples used as a control population were randomly generated. For each pit marked by the operator, the software drew an additional circle as a virtual pit placed at random within 10.0 mm of the original, for which the wall overlap was marked and computed.

### Experiment 2: initial deposition (pairs of pits)

**Fig. 5:**
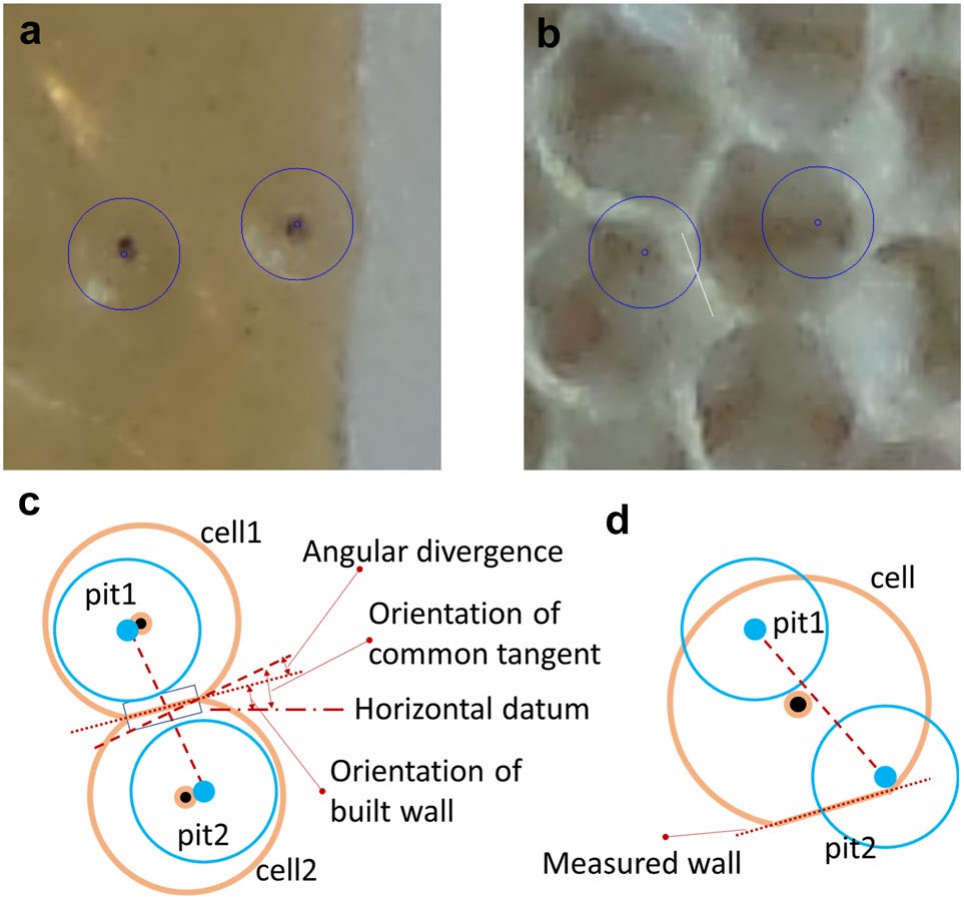
Measurement of a cell wall located between a pit-pair stimulus. (**a**) The marks placed on the photograph recording the initial state of the stimulus, highlighting the location of the pits. (**b**) The photographic record of the same tab after construction had begun, showing the location of the original pits, and the line used to mark the cell wall between the pit centres. (**c**) Diagrammatic representation of the stimulus, the resulting comb, the marks placed by the operator and the resulting angle computed by the software. (**d**) An example of an exception where the nearest wall was not positioned between the pit centres

To measure the association between cell walls and pit-pair stimuli, we used the frame comparison tool FormImageCompare:pitPair() with which we marked the centres of each pit in a pair of depressions. Using these locations, the software calculated the line between the centres, and hence the orthogonal pit common tangent. While viewing the second image, recorded post-construction, we manually marked the line of the wall nearest to the mid-point between the pit centres (Fig. 5).

Samples used as a control population were randomly generated. The software drew an additional pair of pit marks at a random location, orientation and separation (between 5.0 and 6.0 mm centre to centre). The operator then marked a wall found between the two random marks in the same fashion as previously done for real stimuli.

### Experiment 3: initial deposition (V-form)

**Fig. 6:**
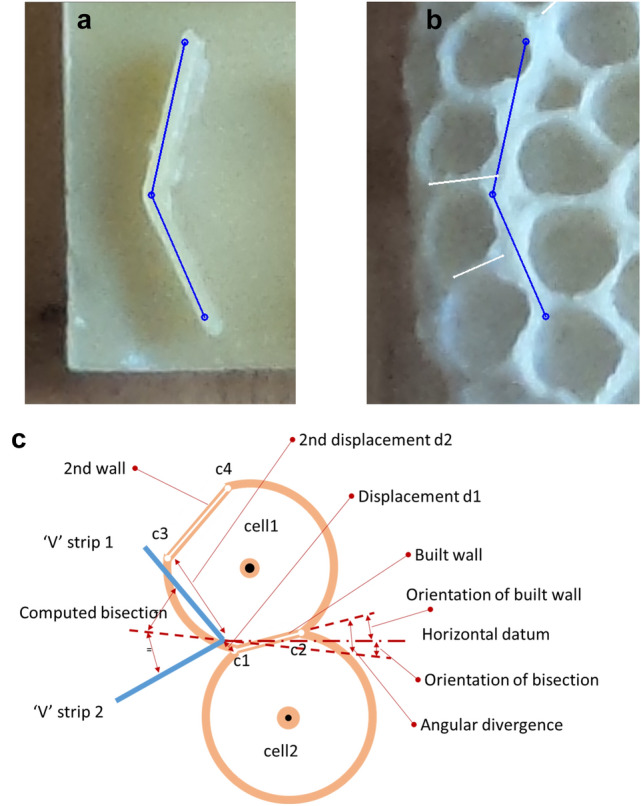
Measurement of a cell wall located at the apex of a V-shaped stimulus. (**a**) The two ends and the apex of the V-shaped stimulus are marked on the photograph of the wax stimulus prior to insertion into the colony. (**b**) The aligned, later-stage image, with the two closest cell walls indicated in white. (**c**) A diagrammatic representation of the V-shape, two cells built close by and the two closest walls. The diagram also shows the measurements computed and exported by the software: the angular difference between the closest wall and the V bisection, and both wall (corner) displacements

The alignment between a V-shaped seed and the associated cell wall was measured using the coordinates of points marked on paired images of each face of the subject comb, recorded before and after construction. Using the tool FormImageCompare:bend(), while viewing the initial image, we marked both ends and the apex of the ‘V’ using which software calculated the orientation of the ‘V’ bisection. While viewing the second, post-construction image, we marked the inter-cell walls that were closest and second closest to the apex (Fig. 6c).

Samples used as a control population were randomly generated. The software drew an additional V mark at a random location, orientation, and splay (between 90° and 152.2°). We then marked the walls associated with the random mark in the same fashion as described for real stimuli.

### Experiment 4: initial deposition (V-form with dual pits)

**Fig. 7:**
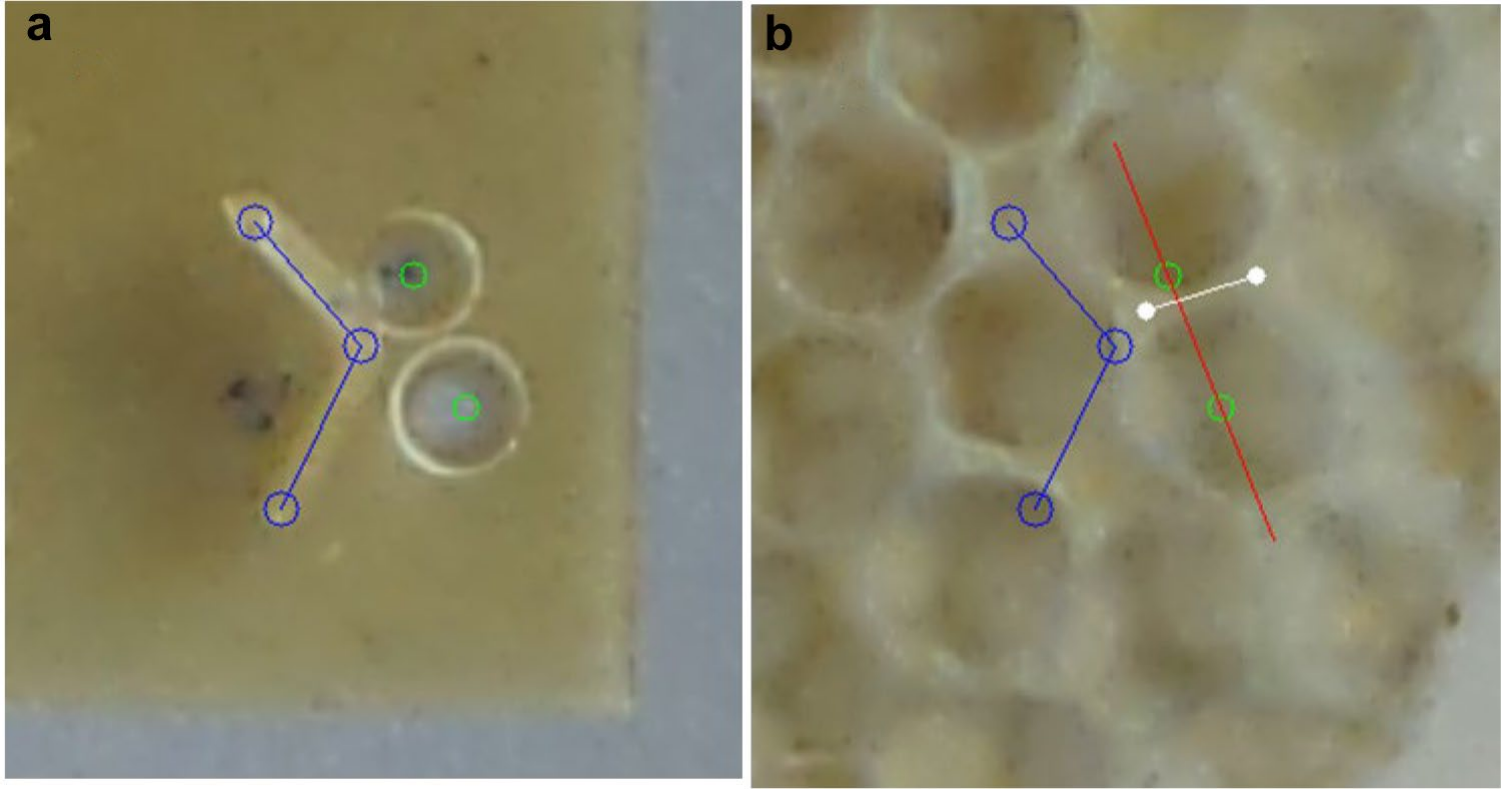
Measurement of a cell wall located at the apex of a combined stimulus including both a V-form and pit-pair. (**a**) Markings on a photograph of the initial stimulus indicate the two ends and the apex of the V-form (blue) and the centre of both pits (green). (**b**) The aligned, later-stage image shows the comb constructed upon the stimulus. The two ends of the cell wall closest to the V apex were marked by the operator and a line was drawn in white by the software. A computer-drawn line (red) connecting the centres of the two pits previously marked by the operator is also shown

For this experiment, stimuli were configured to have both a V-shaped seed and two pit depressions. Measurement of the outcome from these used the same techniques as those described for Experiments 2 and 3. The frame comparison tool FormImageCompare includes a feature to measure ‘bend’. This feature allows the user, when viewing the first image recorded before any comb had been built, to mark the ends of both strips that form the ‘V’ and the apex between. This feature also allows the user to mark the centres of a pair of depressions (Fig. 7a). The software then confirmed the placement of the marks by drawing circles for each mark, with lines joining the ‘V’ locations.

The second image (Fig. 7b), recorded post-construction, was used to mark the line of an inter-cell wall closest to the apex. Two marks placed at the ends of the nearest wall provide the orientation of the wall and, therefore, allow the calculation of the angular divergence between the wall and the V-shape bisection, as well as the divergence between the wall and the pit common tangent.

The ‘V’ stimulus marks allowed the software to calculate the orientation of the bisection line and, from the locations of both pit centres, to calculate the orientation of the pit common tangent. Comparing these values with the orientation of the built wall yielded the divergence of the wall orientation from each of the seed stimuli.

The layout of a V seed, the pit pair, and the cell wall that was marked by the operator on each occasion is shown in Fig. 7.

### Analysis

Data obtained from the images were processed using custom scripts written in R and run within RStudio version 1.3.1093, incorporating R version 3.6.3.

*Experiment 1: initial deposition target (single pits).* The measurement for each sample was the angular overlap between the rim of the pit stimulus and the wall of subsequent cells. This measurement was made for both physical pits offered as stimuli and for randomly placed virtual pits. Comparison between experimental and random populations was made using a Student’s T test, unpaired, two-tailed using the R function t.test() (Fig. 9).

*Experiment 2: initial deposition target (pairs of pits).* The measurement made for each wall between a pit pair was the angular difference between that wall and the theoretical tangent common to the two pits (Fig. 5c). This measurement was made for both physical pits offered as stimuli and for randomly placed virtual pits. Comparison between experimental and random populations was made using a Wilcoxon ranked test with the R function wilcox.test(), as the data were not normally distributed (Fig. 10).

*Experiment 3: initial deposition target (V-form*). Two measurements were made for cell walls close to the apex of the V-form stimulus. The first measurement was the angular difference between a wall and the theoretical line bisecting the V-form (Fig. 6c). The second measurement incorporated the distance from the V-form apex to the closest cell corner (d1) and the distance from the apex to the next-nearest corner (d2; Fig. 6c). These distances were combined to form a metric of proximity, P: with the ratio calculated as *P* = *d*1/(*d*1 + *d*2). Comparisons between experimental and random populations for both metrics were made using Wilcoxon ranked tests with the R function wilcox.test(), as the data appeared not to be normally distributed (Figs. 11 and 12).

*Experiment 4: initial deposition target (V-form plus pairs of pits*). Two measurements were made for cell walls close to the apex of the V-form stimulus. The first measurement was the angular difference between a wall and the theoretical line bisecting the V-form (Fig. 6c). The second measurement was the angular difference between a wall and the common tangent between the pair of pits (Fig. 5c). Comparisons between these dual-stimuli populations and the single stimuli populations obtained from experiments 2 and 3 made using Wilcoxon ranked tests with the R function wilcox.test().

## Results

### Experiment 1: cell wall position was influenced by pit placement

**Fig. 8:**
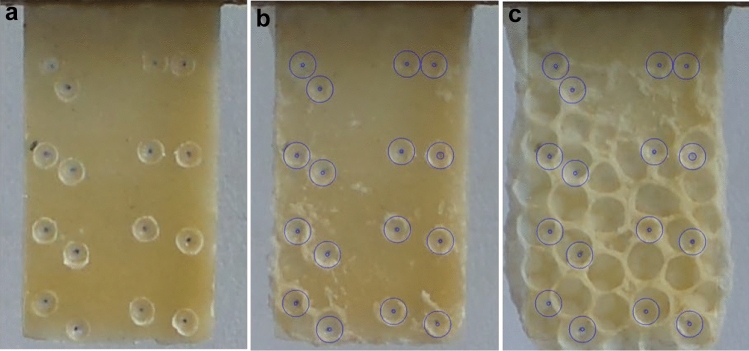
An example of rim depositions which developed into cell walls. (**a**) The stimulus comprising pits pressed into the wax tab. (**b**) Early construction activity comprising wax deposits focussed at the edge of the pit stimuli. (**c**) Following further construction, the early foundations have been built upon resulting in cells with walls coincident with the pit stimuli rims. Panels **b** and **c** show an aligned blend of the original image with that of the later stage and include blue circles, added by the software, that mark the rim of the pit stimuli.

During this experiment, we observed that early activity around the pit stimuli involved wax deposition at a narrow portion of the rim that was extended into a lip encompassing an increasing part of the circumference. Eventually, this foundation was enhanced by the construction effort to become a cell wall. Such interim stages of pit-focussed development were occasionally captured by successive photographs (Fig. 8).

Measurements were taken from 14 frames, each of which carried three wax sheets into which the pit depression stimuli had been pressed. A total of 233 pits and the subsequent beginnings of comb cells were identified and measured on 21 tabs. A further 233 randomly located, virtual pits were created by the software from which the control values were obtained. Data are presented as the mean ± standard deviation throughout.

**Fig. 9:**
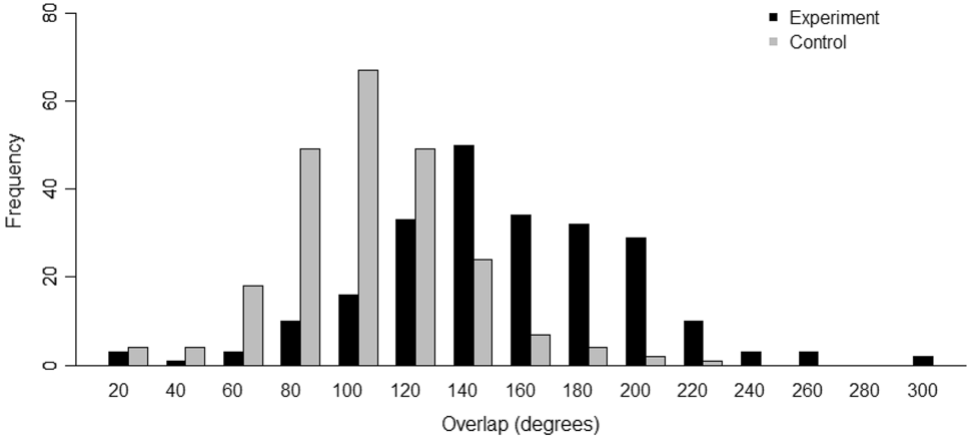
Pit rim-to-wall intersection as the angular overlap between pit rim and cell walls. The distribution of overlap between the rims of pit stimuli and the cell walls built upon them, measured as degrees where the pit rim (either real or virtual) intersected a cell wall. The experimental samples, values shown in black, are for walls around pits pressed into the wax, while the control samples, shown in grey, are for walls coincident with virtual pits placed randomly on the image by the computer software. The higher angular overlap shows that the walls of cells are significantly more aligned with the experimental pit stimuli than with the control samples, the latter being randomly positioned virtual pits

When some comb was built, cell walls overlapped with the rims of the seed pits by 142.4° ± 46.0°, which was significantly greater than the overlap with randomly placed virtual pits (92.3° ± 31.4°; *t*_402_ = 13.6, *P* < 0.00001; Fig. 9) This demonstrates that pit placement influenced the positions of cell walls, supporting our first prediction: that wax deposition will begin at the edges of a stimulus comprising a shallow depression, leading to the eventual location of cell walls. This will occur if one assumes that, upon encountering a sub-cell sized concave shape, a builder’s reaction will be to extend the depression by excavation of wax from the centre.

### Experiment 2: cell wall placement was influenced by pit-pair placement

Measurements were taken from 16 tabs carrying pairs of pits. A total of 66 such pairs and the subsequent beginnings of comb cells were identified and measured. A further 66 virtual pits were created from which the random control values were obtained. For three of these from the experimental set and five from the control set, the built wall extended beyond the centres of the seed pits, and so, these were excluded. Analysis was applied to the remaining 63 and 61, respectively. Data are presented as the mean ± standard deviation throughout.

**Fig. 10:**
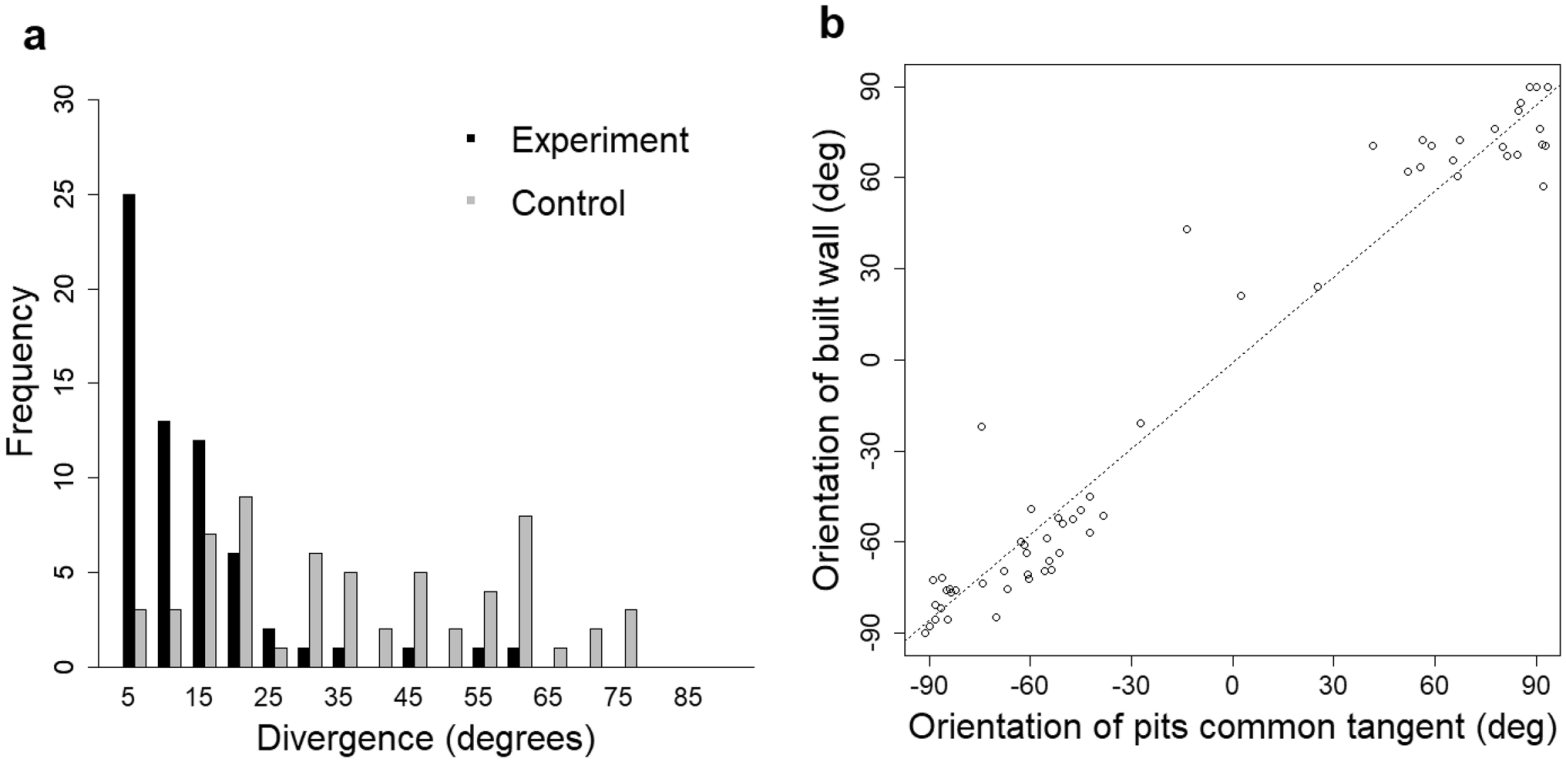
Distributions of divergence between the orientation of the constructed wall and the common tangent of the dual pit stimulus. (**a**) The distribution of experimental and control samples. Walls from the experimental samples were more closely aligned to the common tangent compared with the control samples. (**b**) The orientation of the built wall (*Y* axis) and that of the pit common tangent (*X* axis). The measurement of orientation is based on zero degrees being horizontal. The results show a strong correlation between the two attributes (Spearman’s rank-order correlation, *r*(61) =  + 0.939, *p* < 0.0001), as well as a relational coefficient close to unity (+ 0.942)

When some comb was built, cell walls diverged from the pit common tangent by 9.8° ± 5.1°, which was significantly less than the divergence from the common tangent of randomly placed virtual pits (35.9° ± 15.5°; *W* = 8, *P* < 0.00001; Fig. 10a). This demonstrates that pit placement influenced the positions of cell walls, as predicted by P2 that a stimulus formed from two small depressions will result in a wall aligned to the common tangent between the two pits.

For the experimental set, the orientation of the common tangent lay (clockwise angle from horizontal) compared with that of the built cell wall is shown in Fig. 10b. The orientations were highly correlated (Spearman’s rank correlation: *r*(61) =  + 0.939, *p* < 0.0001) and the relationship between the orientations was close to unity (ratio of orientations of built wall to common tangent = 0.942).

### Experiment 3: cell wall position was influenced by V-strip placement

Measurements were taken from 16 tabs carrying ‘V’ stimuli. A total of 79 such stimuli and the subsequent beginnings of comb cells were identified and measured. A further 79 virtual ‘V’ stimuli were also created, from which the random control values were obtained.

**Fig. 11:**
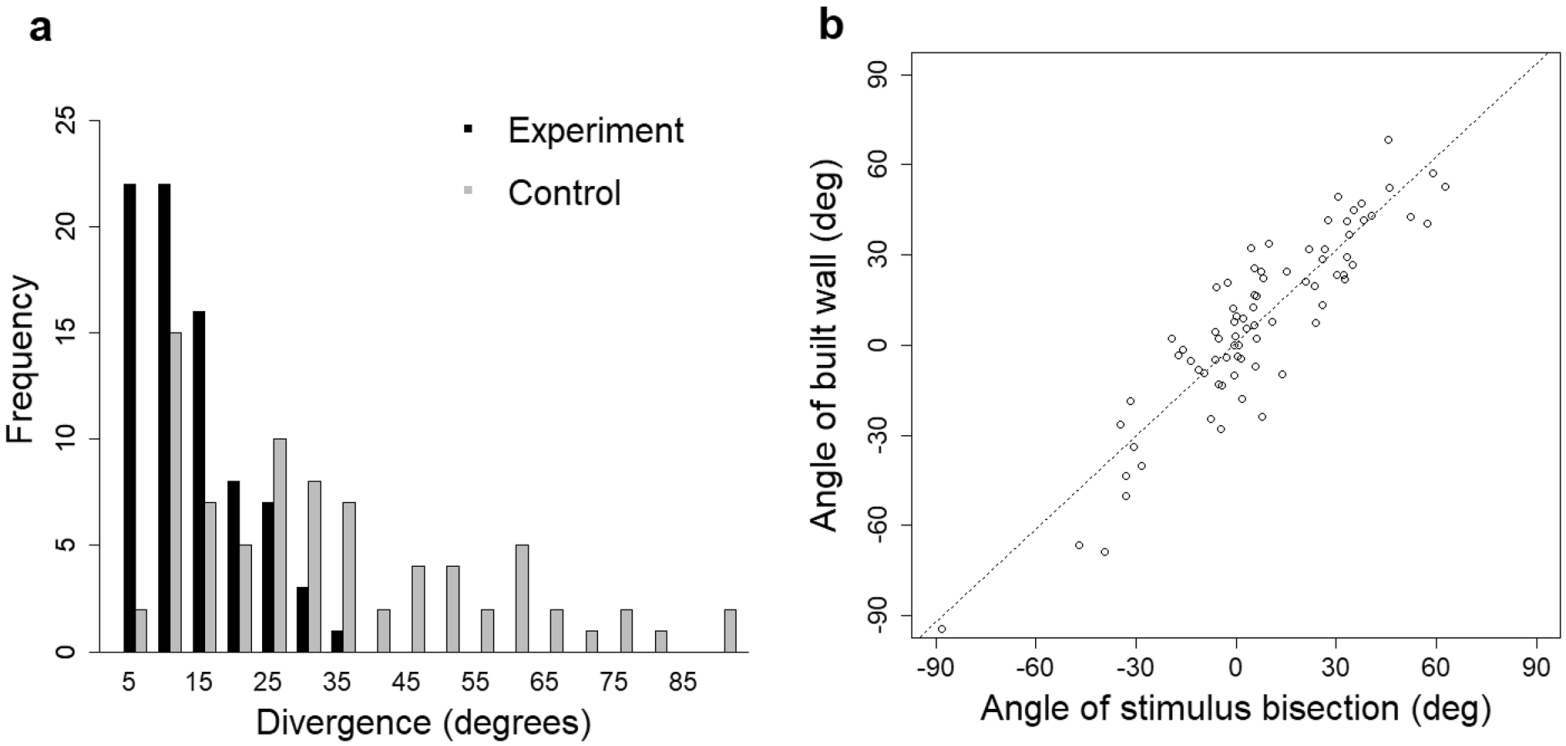
Distribution of angular divergence between the orientation of the constructed wall and the ‘V’ stimulus bisection. (**a**) The distribution of experimental and control samples, with the wall for the experimental samples being more closely aligned to the bisection compared with the control set. (**b**) The orientation of the built wall (*Y* axis) and that of the V bisection (*X* axis). The measurement of orientation was based on zero degrees being horizontal. The results show a strong correlation between the two attributes (Spearman’s rank-order correlation, *r*(77) =  + 0.863, *p* < 0.0001), as well as a near unity ratio between the angle of built wall to stimulus bisection (1.03)

When some comb was built, cell walls diverged from the ‘V’ bisection by 10.5° ± 7.65°, which was significantly less than the divergence from the bisection of randomly placed ‘V’s (30.1° ± 21.2°; *W* = 1215, *P* < 0.00001; Fig. 11a). This demonstrates that ‘V’ strip placement influenced the positions of cell walls, as predicted, that each arm of the V-shape will promote formation of a cells resulting in a cojoined wall at the apex.

The correspondence between the orientation of the ‘V’ bisection and that of the built wall is shown in Fig. 11b.

### Proximity of walls to the apex

**Fig. 12:**
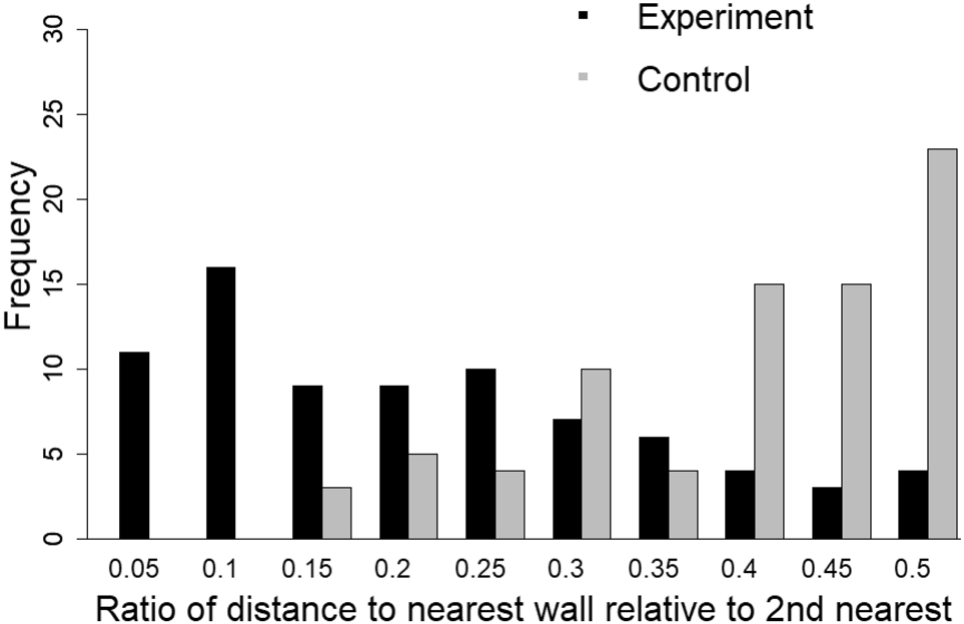
Distribution of relative distances between the apex of the ‘V’ stimulus and the nearest cell corner, expressed as a fraction of the distance from the nearest to the next-nearest corner. The distribution of experimental and control samples, with the wall for the experimental samples being located closer to the apex than for the control set

When some comb was built, the distance between the ‘V’ apex and the nearest wall as a fraction of the distance from that wall to the next-nearest was 0.19 ± 0.13, which was significantly less than that measurement for the apex of randomly placed ‘V’s (0.37 ± 0.11; *W* = 912, *P* < 0.00001; Fig. 12). This demonstrated that ‘V’ strip placement influenced the positions of cell walls as stated by our third prediction: that each arm of the V-shape will promote the formation of a cell, resulting in a cojoined wall at the apex.

During this experiment we observed, but did not measure, the pattern of cells built to the inside of the ‘V’ (Figs. 6b and 7b). This concavity typically resulted in the construction of a cell utilising the internal surface of ‘V’ stimulus, close to the apex, as part of the cell, the additional walls being built from the stimulus to complete the enclosure.

### Experiment 4: cell wall position was preferentially influenced by pit-pair placement

**Fig. 13:**
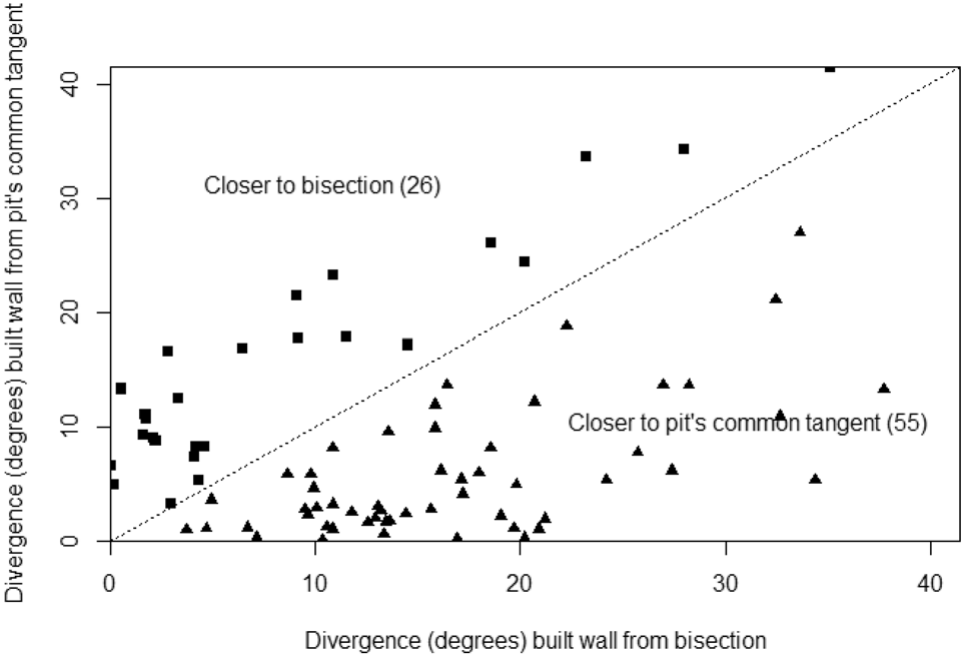
Divergence of the built wall from each of the two potential guides, V bisection and pit common tangent, measured for hybrid stimuli. The division line separates the graph area into regions closer to one influence than the other. Of the population of 81, 55 walls (shown as triangles) were aligned more closely to the pit common tangent compared with 26 (shown as squares) aligned closer to the V bisection line. Alignment of the pits had a greater influence than the V-shape had on the orientation of > 2/3 of the measured walls

**Fig. 14:**
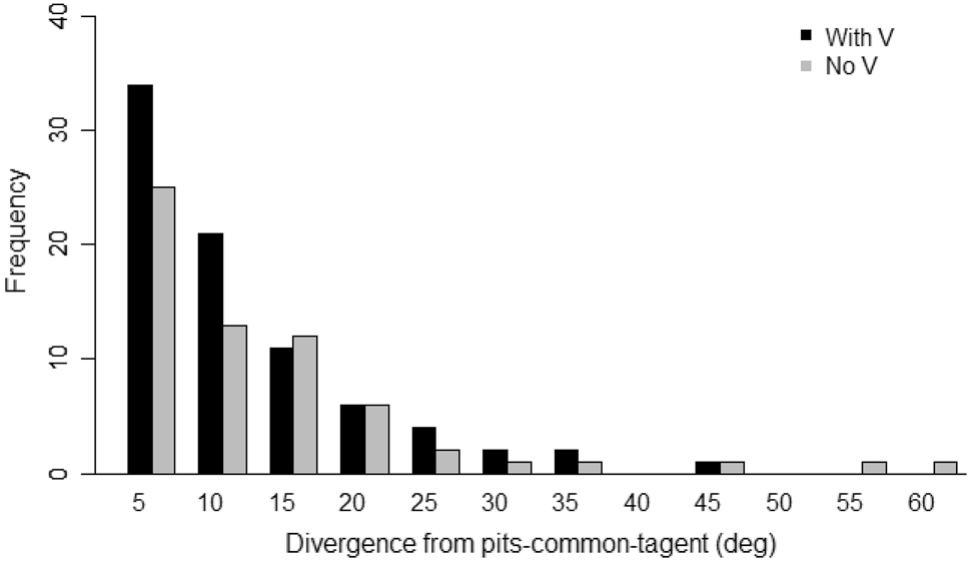
The distribution of divergence between built walls and the pit-pair common tangent. The figure depicts compound stimuli comprising both a V-shape and misaligned pair of pits. The two sets of samples are for stimuli including a ‘V’ form and those without (from experiment 3). The distributions show little difference, suggesting that the additional ‘V’ stimulus has less influence over the orientation of the cells and walls than the pit pairs

**Fig. 15:**
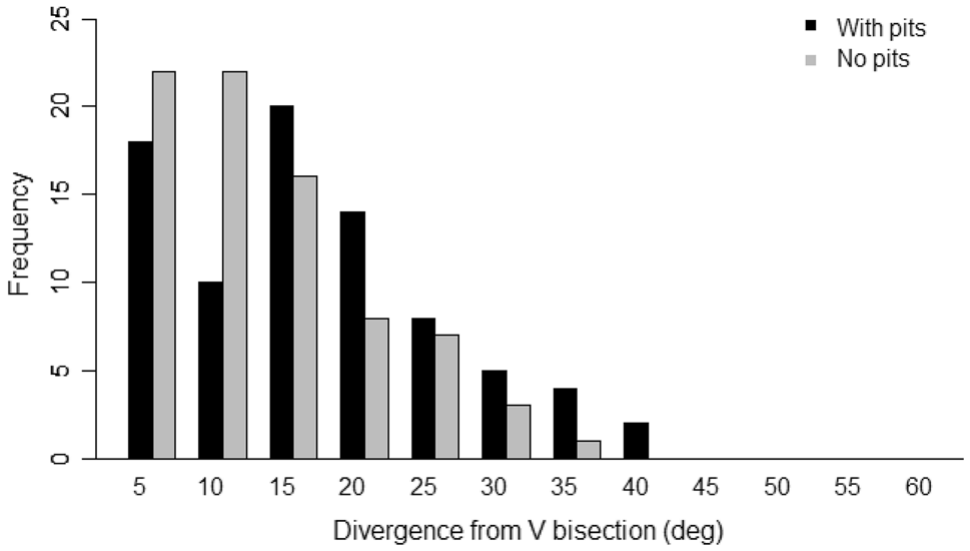
The divergence between built walls and the V stimulus bisection. The figure depicts compound stimuli comprising both a V-shape and misaligned pair of pits. The two sets of samples are for stimuli including a pair of pits and those without (from experiment 2). The distribution of divergences for walls built in the presence of combined stimuli differs from that for walls built on stimuli comprising only a ‘V’ form. This suggests that the influence of pit pairs over the orientation of cell walls is greater than the influence due to the ‘V’ stimulus

Measurements were taken from 81 V-shaped stimuli adorned with pits. When comb was built on the combined ‘V’ and pit stimuli, 55 built walls, from a sample of 81, were closer to the alignment of the pit common tangent than to the ‘V’ bisection (Fig. 13). The cell walls diverged from the pit common tangent by 8.9° ± 8.7°, which was significantly less than the divergence from the ‘V’ bisection (14.0° ± 9.2°, *P* = 0.00005; Figs. 14 and 15). This demonstrated that pit placement influenced the positions of cell walls more than did the ‘V’ strip.

Comparing the divergence from the pit common tangent with or without the additional ‘V’ stimulus, this experiment (8.9° ± 8.7°, Fig. 14) was not significantly different from experiment 3 (10.3° ± 11.7°, Fig. 11A, and Fig. 14; *P* = 0.72). This demonstrated that in the presence of pits, the ‘V’ strip placement had no more influence over the positions of cell walls than may be expected by chance.

Comparing the divergence from the ‘V’ bisection with or without the pits as an additional stimulus, this experiment (14.0° ± 9.2°, Fig. 15) was significantly different from experiment 2 (10.5° ± 7.6°, Fig. 10A and Fig. 15; *P* = 0.01). This demonstrated that in the presence of the ‘V’ strip, the addition of pits had more influence over cell wall position than may be expected by chance.

## Discussion

Many scholars have described the form of completed cells and the architecture of comb, but few have addressed the early stages of construction, the topic of this article (Huber 1814; Darchen 1959). The latter described in detail the sequence of actions that resulted in the construction of two rows of cells. According to these observations, the first stage of cell construction involved a single worker focussing her efforts on a small depression in a wax deposit, extending it by the removal of wax. Huber also noted that *“..the block itself was not of a sufficient length to complete the diameter of the cell. So the bees continued to increase its size*” (1814). Furthermore, in his description of the beginnings of the second row of cells, Huber observed that the base of a new cell was started by extending the surface formed by a valley at the junction between two extant cells. Such observations, together with the possibility that stigmergy directed the bees’ actions, helped guide the development of our hypotheses and our results provide confirmatory evidence for these descriptions.

In addition to this description of early stage cell construction, attempts have also been made to model the steps taken by workers to build comb (Nazzi 2016; Narumi 2018). For example, Nazzi (2016) based his model on the initiation of a cell base at the niche between two extant cells. The results of experiment 1 support these assumptions, showing that a depression, or pit, will act as an instigating cue for the builder whereby it treats the pit as the beginning of a cell, requiring enlargement. This results in the initiation of wax deposition by the builder. The results of experiments 1 and 2 demonstrate that the bees deposit wax at the edges of the concave stimulus. These deposits, following some enlargement, become cell walls. This empirical outcome supports Huber’s further observations that the initial shallow depressions, while still being enlarged, are worked by other bees that take wax scales “ … and apply them upon the edges so as to lengthen them” (Huber 1814). Eventually, these edges became cell walls. Nazzi (2016) also described the early construction process, stating that construction of the cell walls is initiated when the cell base reaches the size of the cell diameter.

Another previous model characterised the early stages of cell formation as an attachment-excavation, where individual actors carve semi-circular cavities within a body of randomly deposited wax to leave a residue similar to natural formations (Narumi 2018). In this model, inter-cell shapes arise through rules that govern the behaviour of the excavators, whereas, here, we posit the mechanism to be one of targeted depositions around a depression; with wax being deposited only where it is needed. Focused placement of wax rather than bulk deposition and subsequent erosion to form the shape of a cell would seem to require less material. One such mechanism to guide these targeted actions might be stigmergy.

A point of commonality with Narumi is, however, that the construction method includes an erosion mechanism. The circular, two-dimensional or spherical, three-dimensional shape of early stage cells is likely formed by the behaviour described by Martin and Lindauer (1966), involving the envelope prescribed by a bee’s mandibles through the movement of her head, articulated at her neck. This mechanism is also assumed by Narumi (2018) to serve as the basis of the shape of excavation.

In summary, during the early stages of cellular construction, bees seek a concave feature to be enlarged and surrounded by the beginnings of a wall, or walls. Our results confirmed our predictions that bees will deposit wax at the edges of a shallow depression, that a shared cell wall will form at the mid-way point between two shallow depressions as this wax is deposited, and that two such depressions joined at a V-shape will cause two cells to be formed with a new wall built at the cell intersection. The tessellation of cells in the characteristic hexagonal layout, shown to be optimal by Lagrange (1773), requires any additional cell to be placed exactly between two extant cells (or three cells, when considering the 3-dimensional nature of double-sided comb). The results presented here show how construction workers can be guided simply by the current form of extant comb to correctly locate new cells; cells that begin as circular (spherical section) becoming hexagonal following further manipulation (Pirk et al. 2004; Hepburn et al. 2007; Gallo et al. 2022). Thus, the comb structure can be built by several individuals, each independently responding to the form of the workpiece. Each worker need only react in a fashion that is appropriate to the present perceived conditions in their immediate locale. Discrete stigmergy describes just such a rule-based association of actions to be taken and the prevailing conditions that stimulate them (Theraulaz and Bonabeau 1999). Stigmergy therefore represents a candidate mechanism that underlies the coordination of comb building by honeybees. In this paper, we have considered only the early stages of cell construction, and more work remains to characterise the formation of a whole cell, let alone the entire comb. Further experiments concerning the reactions of bees to intermediate- or late-staged cell forms will be required to provide a full explanation of cell construction, and to determine whether and to what degree this process is governed by stigmergy.

## Data Availability

The data that support the findings of this study are openly available in https://github.com/VinceGalloQMUL/honeycombThesisRepo.
